# When do the pathological signs become evident? Study of human mesenchymal stem cells in MDPL syndrome

**DOI:** 10.18632/aging.206159

**Published:** 2024-11-26

**Authors:** Spitalieri Paola, Guerrieri Lara, Murdocca Michela, Di Cesare Silvia, Maccaroni Serena, Pecorari Rosalba, Nardone Anna Maria, Candi Eleonora, Colasuonno Fiorella, Gori Giulia, Traficante Giovanna, Novelli Giuseppe, Sangiuolo Federica

**Affiliations:** 1 Department of Biomedicine and Prevention, University of Rome Tor Vergata, Rome, Italy; 2 Department of Systems Medicine, University of Rome Tor Vergata, Rome, Italy; 3 Department of Experimental Medicine, TOR, University of Rome Tor Vergata, Rome, Italy; 4 Medical Genetics Unit, Fondazione Policlinico Tor Vergata, Rome, Italy; 5 Department of Science – LIME, Roma Tre University, Rome, Italy; 6 Meyer Children’s Hospital IRCCS, Florence, Italy

**Keywords:** MDPL syndrome, MSCs, aging, hiPSCs, POLD1 gene

## Abstract

Aging syndromes are rare genetic disorders sharing the features of accelerated senescence. Among these, Mandibular hypoplasia, Deafness and Progeroid features with concomitant Lipodystrophy (MDPL; OMIM #615381) is a rare autosomal dominant disease due to a *de novo* in-frame deletion in *POLD1* gene, encoding the catalytic subunit of DNA polymerase delta.

Here, we investigated how MSCs may contribute to the phenotypes and progression of premature aging syndromes such as MDPL.

In human induced pluripotent stem cells (hiPSCs)-derived MSCs of three MDPL patients we detected several hallmarks of senescence, including (i) abnormal nuclear morphology, (ii) micronuclei presence, (iii) slow cell proliferation and cell cycle progression, (iv) reduced telomere length, and (v) increased levels of mitochondrial reactive oxygen species (ROS). We newly demonstrated that the pathological hallmarks of senescence manifest at an early stage of human development and represent a warning sign for the progression of the disease.

Dissecting the mechanisms underlying stem cell dysfunction during aging can thereby contribute to the development of timely pharmacological therapies for ameliorating the pathological phenotype.

## INTRODUCTION

A thorough understanding of the mechanisms of premature-aging syndromes is essential for developing novel and effective therapeutic strategies. The absence of suitable *in vitro* cell models that accurately reflect human disease phenotypes has hindered the understanding of the molecular mechanisms underlying aging and disease development.

Over the past decade, the groundbreaking discovery of reprogramming somatic cells into human induced pluripotent stem cells (hiPSCs) has opened up unprecedented opportunities for translating laboratory discoveries into novel therapies, bringing us closer to realizing effective stem cell-based medicine. The ability to generate patient-specific hiPSCs, combined with advancements in stem cell differentiation techniques, presents a powerful opportunity to develop phenotypically complex disease models. These models hold significant potential for an early understanding of the onset of the pathology during tissue development, which is reflected in effective diagnosis and drug screening to achieve a personalized therapy.

Premature-ageing syndromes represent rare genetic disorders that exhibit characteristics reminiscent of accelerated aging. To date, more than one hundred syndromes displaying premature ageing features have been documented worldwide, with an estimated incidence of approximately 1 in 50,000 [1]. These conditions can originate from abnormalities in nuclear lamina architecture, or mutations in genes crucial for DNA repair and genome stability maintenance, or even connective tissue alterations and mitochondrial dysfunction, among others [2].

Within this framework, Mandibular hypoplasia, Deafness and Progeroid features with concomitant Lipodystrophy (MDPL; OMIM #615381) is a rare autosomal dominant disease. It is a systemic disorder characterized by notable loss of subcutaneous fat, distinct facial features, and metabolic irregularities such as insulin resistance and diabetes mellitus. The molecular basis of MDPL is a recurrent *de novo* in-frame variation in *POLD1* gene (c.1812_1814delCTC, p. Ser605del) [3–5], which encodes the catalytic subunit of DNA polymerase delta, crucial for lagging strand DNA synthesis during replication. MDPL patients show an abnormal distribution of adipose tissue by either complete or partial absence of white adipose tissue (WAT), which may occur in conjunction with adipose mass redistribution. Other established cellular hallmarks of aging include reduced autophagy, aberrant activation of immune cells, cellular senescence, inflammation, and diminished renewal potential and functional plasticity of adipose tissue, due to the functional decline of adipose-derived stem cells, such as mesenchymal stem cells (MSCs) [6, 7].

MSCs can be isolated from various tissues, including bone marrow, adipose tissue, cartilage, dental pulp, umbilical cord blood, and placenta. These cells have the capacity to differentiate into multiple cell lineages, such as osteocytes (bone), chondrocytes (cartilage), adipocytes (fat), and even neuron-like cells. MSCs represent tissue-specific progenitor cells from which MDPL targets disease tissues, particularly skeletal, adipose, and muscular tissues.

Isolating and propagating adult human stem cells (MSCs) from disease-bearing tissues sometimes can be complex and challenging. Due to these unique qualities, hiPSCs present a viable alternative for studying multipotent stem cells, like MSCs.

Stem cell senescence is a cellular response to endogenous or exogenous stresses that restrict their proliferation and function [8]. Often regarded as a hallmark of aging, MSC senescence can be triggered by various stimuli, such as oxidative stress due to reactive oxygen species (ROS) accumulation, UV radiation, chemical exposure as drugs or toxins, including heavy metals and industrial chemicals, or replicative exhaustion. Senescent cells accumulate in aged tissues [8, 9] and contribute to the development of age-related diseases. Understanding the adverse impacts of senescent MSCs on tissue repair and regeneration is crucial for developing therapeutic interventions.

Over the last three years, we have studied various aspects of the cellular pathology observed in MDPL, such as nuclear alterations, impaired cell growth, cellular senescence, impaired repair of DNA double-strand breaks, presence of micronuclei and mitochondrial dysfunction [10, 11].

Despite the plasticity of fibroblasts in contributing to the understanding of the cellular phenotype observed in MDPL, for the purposes of a possible effective clinical translation, rigorous verification in organotypic cultures, xenografts and organoids derived from pluripotent cells is necessary [12].

We, therefore, studied patient-derived induced pluripotent stem cells (hiPSCs) obtained from MDPL patients by dedifferentiating fibroblasts *in vitro* [13] and committed them into mesenchymal (mesodermal) stem cells (MSCs), which are also able to self-renew and differentiate in mesodermal tissues. Our hypothesis is that MSCs contribute to the phenotypes and progression of premature-ageing syndromes like MDPL.

**Figure 1:**
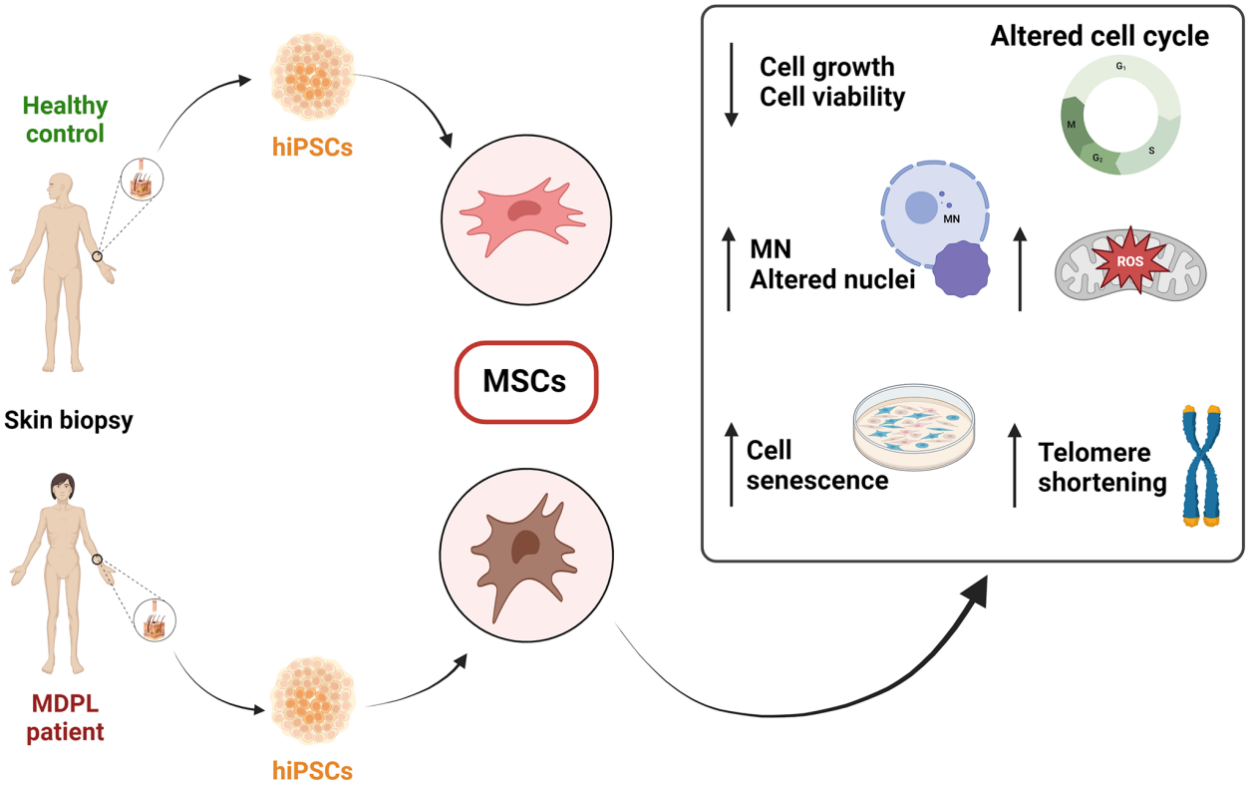
Schematic overview of the findings from the characterization of mesenchymal stem cells (MSCs) derived from hiPSCs in MDPL patients and healthy donors.

In this study, we report the *in vitro* characterization of mesenchymal stem cells (MSCs) hiPSC derived from three patients with clinical features of MDPL syndrome and two healthy donors to better elucidate their cellular phenotype. In fact, some senescent characteristics appear already evident in hiPSCs and even more in MSCs. Specifically, MDPL-hiPSCs show some abnormalities in nuclear morphology and the presence of micronuclei, never described before in this kind of cells, while hiPSC-derived MSCs, besides these aspects even more marked, exhibit a slowed cellular proliferation and cell cycle progression as well as reduced telomere length and higher levels of mitochondrial reactive oxygen species (ROS) (Figure 1). Also, once hiPSCs have been committed to differentiate into MSCs, FACS analysis revealed a reduced efficiency of differentiation into CD44, CD90 and CD73 positive cells.

The comprehensive nature of these experiments represents a groundbreaking effort, as such evaluations have not been previously conducted in the context of MDPL syndrome.

## RESULTS

### MDPL hiPSCs display a marked heterogeneity in the differentiating capacities

**Figure 2:**
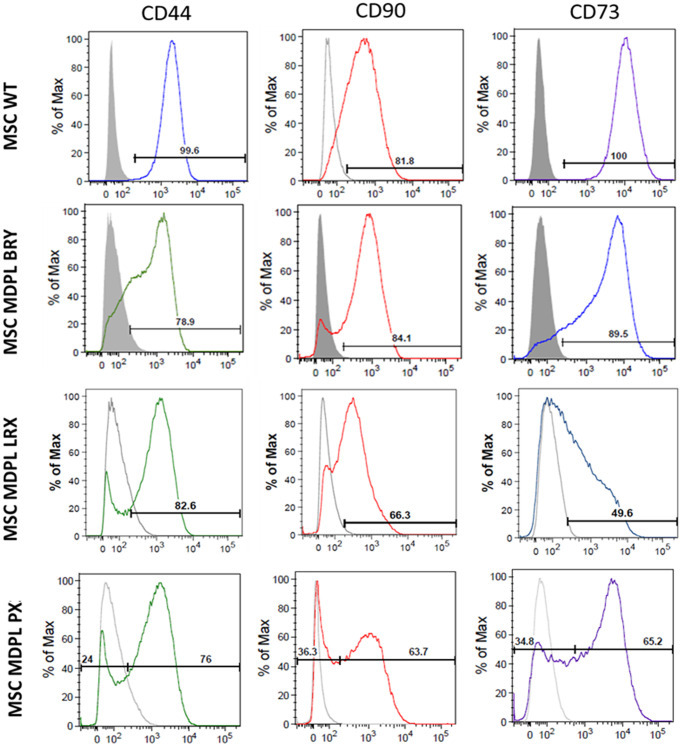
**Expression of MSC markers in hiPSC-derived MSCs.** Representative histograms of the flow cytometric analysis of the markers CD44, CD90 and CD73 in WT and MDPL MSCs. Grey solid histograms represent the fluorescence of isotype controls, while the empty black histograms represent the specific fluorescence of the indicated MSC markers. The MSC WT histogram is representative of one of two healthy controls because values were comparable. The analyses were performed at passage 3.

Once we obtained hiPSCs from MDPL and healthy individuals (WT) (Supplementary Data and Supplementary Figure 1) [14], we differentiated them into mesenchymal cell lines by optimizing differentiation protocols [15–17]. To this purpose, we treated embryoid bodies (EBs) derived from hiPSCs, with all-trans retinoic acid (RA) at concentrations of 10 and 0.1 µM. After 20 days of culture [16–18], the cellular [16–18] morphology began to change exhibiting a spindle shape with a small cell body and a few long thin processes as well as a large nucleus with a differentiated nucleolus. Upon undergoing 2–3 passages, the differentiation efficiency of MSCs is assessed by quantifying specific surface markers. Reliable MSC differentiation should exhibit greater than 80% efficiency for mesenchymal surface markers CD73 (ecto-5′-nucleotidase), CD90 (Thy1), and CD44 (H-CAM) by flow cytometry analysis [16–18]. Additionally, cells are evaluated for the absence of surface markers indicative of a hematopoietic phenotype, such as CD14, CD19, and CD45, and are expected to show less than 1% expression efficiency for these markers. As observed, MSCs surface markers are consistently expressed (>80% positivity) in WT hiPSC-derived MSCs, while the MDPL lines show reduced expression of CD90, CD44 and CD73 compared to WT MSCs, more evident in the LRX and PX MDPL lines (Figure 2). These data evidenced a marked heterogeneity in the differentiating capacities from hiPSCs to MSCs. Parallelly, the absence of hematopoietic lineage is confirmed by the low percentage (<0.7%) of cells positive for CD14, CD19 and CD45 in all lines of WT and MDPL (data not shown).

### Polδ protein expression in MDPL and WT MSCs

**Figure 3:**
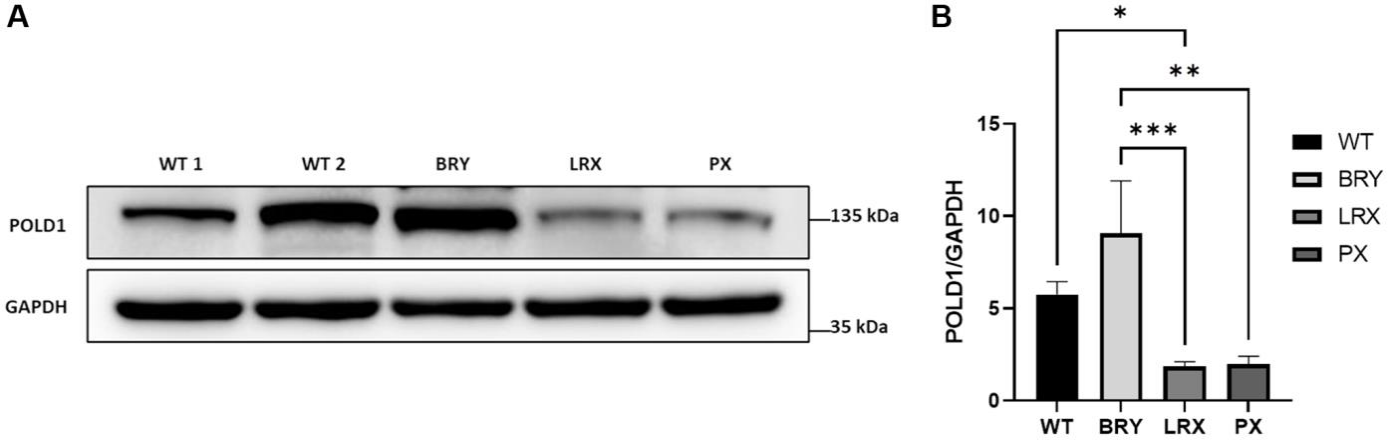
**Analysis of POLD1 protein expression levels in MDPL and WT-MSCs.** (**A**) Protein extracts of MDPL-MSCs, compared with healthy controls. (**B**) Densitometric analysis of POLD1 protein normalized on GAPDH levels; ^*^p < 0.05, ^**^p < 0.01, ^***^p < 0.001, by one-way ANOVA test.

Once we confirmed the heterozygous c.1812_1814delCTC variant in MSC from MDPL patients (i.e., BRY, LRX and PX) by Sanger sequencing of *POLD1* gene, we evaluated DNA polymerase δ protein expression levels by Western blot analysis to assess the phenotype of POLD1 p.Ser605del variant at protein level in different patients (Figure 3A). Densitometric analysis (Figure 3B) revealed decreased level of Polδ protein in LRX and PX MSCs with respect to the average of the MSCs from healthy controls (WT). Moreover, this difference is statistically significant only for LRX-MSCs (^*^p < 0.05). In contrast, BRY-MSCs showed an upregulation of POLD1 protein expression levels, although not significant. Surprisingly, even though our patients share the common POLD1 pSer605del variant, our results revealed a strong significant difference in POLD1 protein expression in BRY-MSCs compared to LRX and PX patients (^***^p < 0.001 and ^**^p < 0.01 respectively), reflecting an individual heterogeneity among patients.

### MDPL MSCs display hallmarks of aging

**Figure 4:**
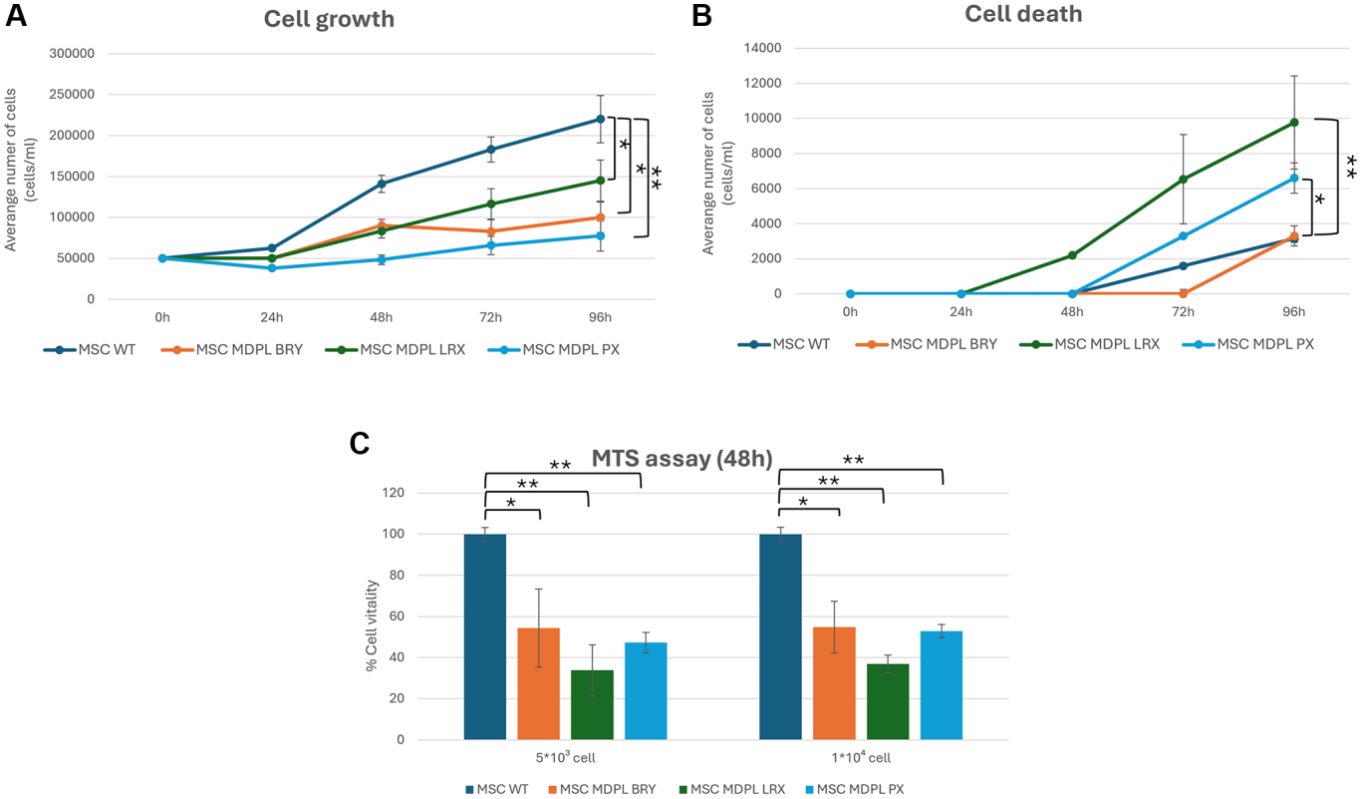
**Cell viability of MSCs.** (**A**) Population doubling levels of WT (blue) and MDPL MSCs (orange, green and light blue) from 0 to 96 hours. (**B**) Cell death count in MSCs of WT and MDPL MSCs from 0 to 96 h. (**C**) MTS assay, histogram representing the percentage of cell viability at 48 h of WT and MDPL MSCs at 5 × 10^3^ and 10 × 10^3^ cell seeded. The WT data are derived from the mean of the values obtained from the two healthy control samples. Error bars indicate the standard deviation ± standard error of the mean (SEM). ^*^p < 0.1, ^**^p < 0.01, by one-way ANOVA test.

Given our previous results obtained on MDPL primary cells [10], we decided to investigate proliferation capacity and cellular death, two characteristics playing a pivotal role in human aging [19]. Effectively, MDPL cells exhibited a significantly slower rate of growth and proliferation compared to control cells over a 96-hour period. This was evident at all time points examined (0, 24, 48, 72, and 96 hours). Specifically, at the 96-hour mark, PX, LRX, and BRY had significantly lower cell counts than the WT: a reduction of the proliferation respectively of around 64.8%, 34.1% and 54.4% was observed (Figure 4A). Additionally, LRX and PX showed a marked increase in cell death compared to the control, while BRY exhibited a similar trend to the control (Figure 4B). These differences were statistically significant (^*^p < 0.1; ^**^p < 0.01). To further validate these findings, an MTS assay was conducted at two different cell seeding densities. The assay shows a reduction of cell viability of around 45% in BRY patient, 65% in LRX patient and 50% in PX patient with respect to both WT expressed as 100% (^*^p < 0.1; ^**^p < 0.01) (Figure 4C).

### MDPL MSCs show increased nuclear anomalies and micronuclei (MNs) presence

**Figure 5:**
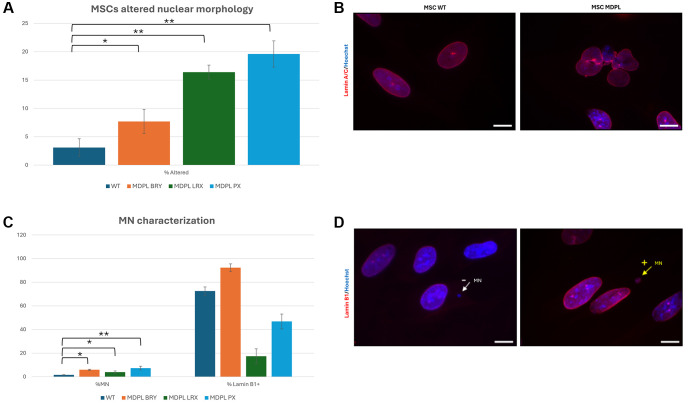
**MSCs altered nuclear morphology analysis.** (**A**) Histogram representing the percentage of aberrant nuclear morphology. (**B**) A representative image of MSCs WT and MDPL obtained by immunofluorescence for anti-Lamin A/C antibody (red). (**C**) Percentage of micronuclei (%MN) and percentage of Lamin B1 positive (% Lamin B1+) MN in MSCs MDPL and WT. (**D**) Representative immunofluorescence images showing the presence of micronuclei (MN) positive (arrow yellow) or negative (arrow white) for anti-Lamin B1 antibody (red) in MDPL cells. Hoechst nuclear staining (blue). Scale bar 100 µm. The WT data are derived from the mean of the values obtained from the two healthy control samples. Experiments were carried out in triplicates; 300 nuclei were counted per individual. Error bars indicate the standard deviation ± standard error of the mean (SEM). ^*^p < 0.1, ^**^p < 0.01 by one-way ANOVA test.

HiPSCs-derived MSCs from MDPL patients recapitulate the cellular features observed in senescent cells. In fact, we noted nuclear invaginations, large protrusions (blebs) and doughnut-shaped nuclei, with a frequency higher than in WT-MSCs (3.1% in WT obtained as a median value, compared to 16.4% in LRX, 7.7% in BRY and 19.6% PX; ^*^p < 0.1, ^**^p < 0.01) detected by anti-Lamin A/C antibody (Figure 5A, 5B). These features have already been previously described in fibroblasts of MDPL patients [10, 20] and are signs that are usually present in cellular models obtained from patients with progeroid disorders. Moreover, nuclear staining with Hoechst revealed the presence of micronuclei up to 3 times more in MDPL cells (LRX 3.9%, BRY 5.9% and PX 7.3%) than in WT ones (median values: 1.6%) (^*^p < 0.1, ^**^p < 0.01) (Figure 5C). Immunostaining with anti-Lamin B1 antibody (Figure 5D) revealed in MDPL MSCs, 17.4% of micronuclei are Lamin B1 positive in LRX, 46.8% in PX and 92.3% in BRY.

### Karyotype analysis

Both hiPSCs (data not shown) and MSCs underwent karyotype analysis to determine chromosomal identification and any abnormalities involving chromosome number as well as changes in chromosome structure and stability. G-banded analysis of MDPL and WT metaphase chromosomes revealed a normal karyotype of 46 chromosomes in all lines analyzed (Supplementary Figure 2).

### MDPL MSCs reveal a slowed cell cycle, a senescent phenotype and telomere shortening

**Figure 6:**
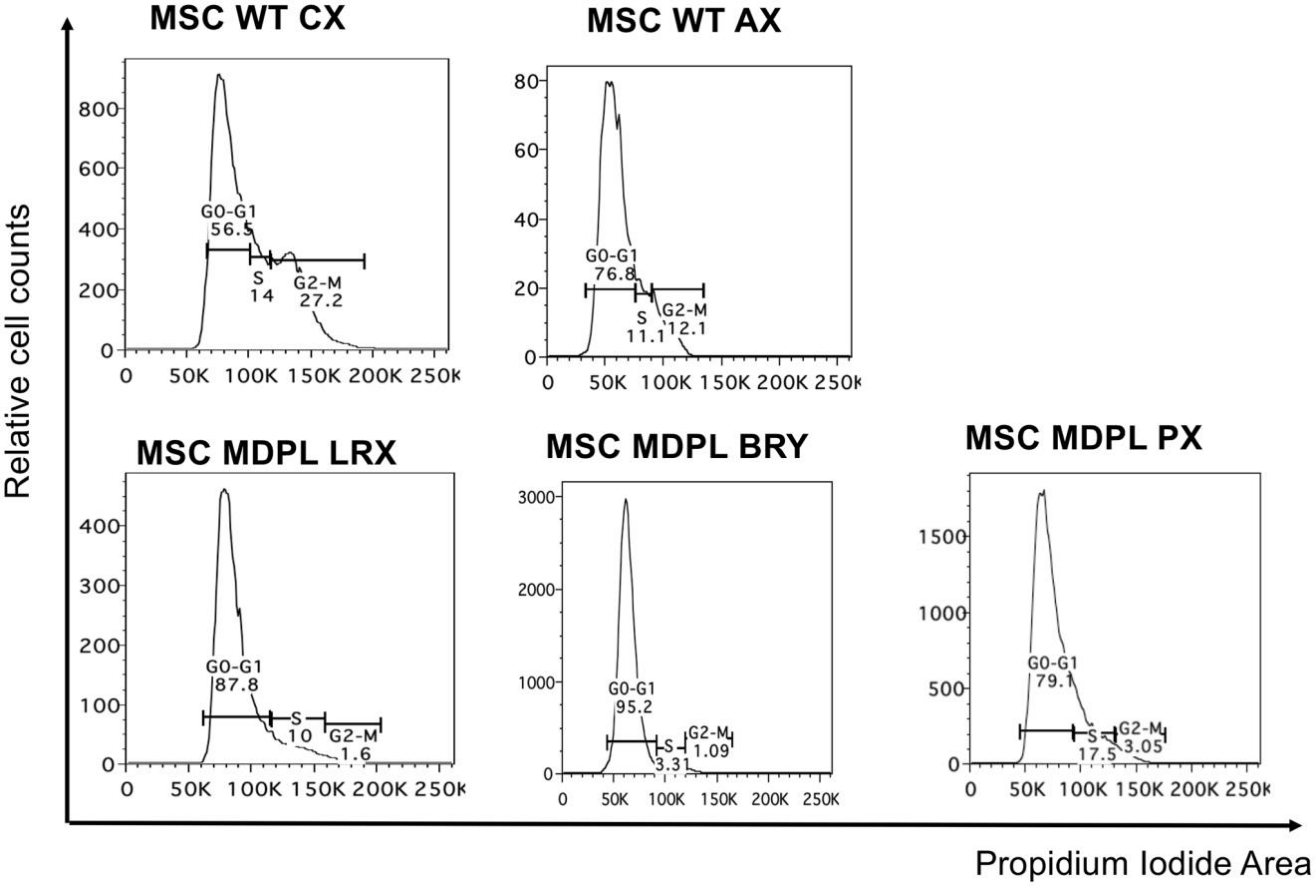
**Cell cycle analysis.** Histogram plots showing cells in G0/G1, S and G2/M phases of the cell cycle performed with the DNA intercalating dye propidium iodide.

MSCs cell cycle was evaluated by flow cytometric analysis after Propidium iodide (PI) staining. Results are shown in Figure 6 showing a decrease of MDPL cells in G2/M phase and an increase in G0/G1 phase with respect to WT ones. Specifically, LRX shows 1.6% of cells in G2/M phase, BRY 1.09% and PX 3.05% with respect to CX 27.2% and AX 12.1% of WT cells. We observed an opposite trend in G0/G1 phase, revealing a quiescence state in MDPL lines (87.8% LRX, 95.2% BRY, 79.1% PX) compared to WT ones (in a range from 56.5% to 76.8%) (Figure 6).

**Figure 7:**
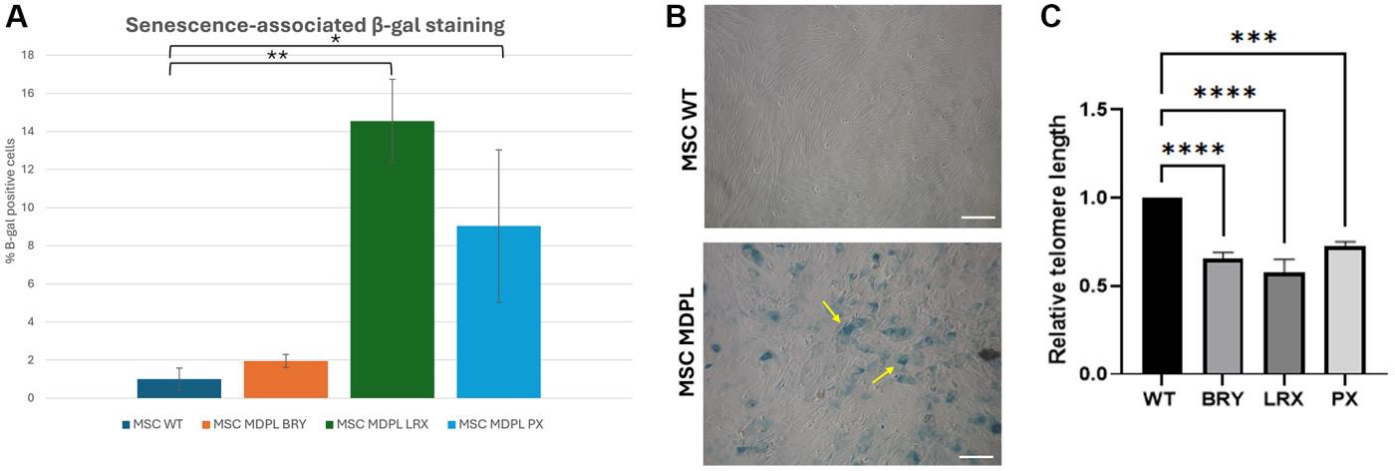
**MDPL MSCs senescence and telomere shortening.** (**A**) The histogram shows the average percentage of β-galactosidase-positive cells in WT and MDPL mesenchymal stem cells. (**B**) Representative image of senescence-associated β-galactosidase assay. A greater amount of intensely positive blue cells is displayed in MDPL-MSCs than in WT controls. Arrows indicated altered cell shape. Scale bar = 200 µm. Experiments were carried in triplicates; 100 nuclei were counted per individual. Error bars indicate the standard deviation ± standard error of the mean (SEM), ^*^p < 0.1, ^**^p < 0.01. (**C**) Relative telomere length was evaluated by real-time q-PCR in MSCs MDPL and WT. Experiments were carried in triplicates. Error bars indicate the standard deviation ± standard error of the mean (SEM). ^***^p < 0.001, ^****^p < 0.0001 by one-way ANOVA test.

To deepen this aspect, a β-galactosidase assay was performed comparing WT and MDPL-MSCs. A higher number of senescent cells were detected in MDPL-MSCs BRY (1.94%), LRX (14.5%), and PX (9.04%) when compared to WT β-galactosidase-positive cells (0.9% ± 0.58, average of the two WT controls) (^*^p < 0.1, ^**^p < 0.01) (Figure 7A, 7B). Morphological analysis revealed MDPL-MSCs, which appeared more enlarged, lost their spindle-shaped characteristics, and flattened. Instead, normal morphology of MSCs WT in cell culture was characterized by a spindle shape with a small cell body and a few long thin processes as well as a large nucleus with a differentiated nucleolus (Figure 7B).

Since telomere shortening is another important trigger of cellular senescence associated to cell cycle, we evaluated the relative telomere lengths of MDPL MSCs with respect to healthy controls. In Figure 7C, we evidenced a significant reduction in all MDPL MSCs compared to WT, ^***^p < 0.01, ^****^p < 0.001). These data suggest that MDPL MSCs suffered from an evident telomeric shortening, which could represent one of the causes of the restricted proliferative capacity.

### Mitochondrial dysfunction in MDPL MSCs

**Figure 8:**
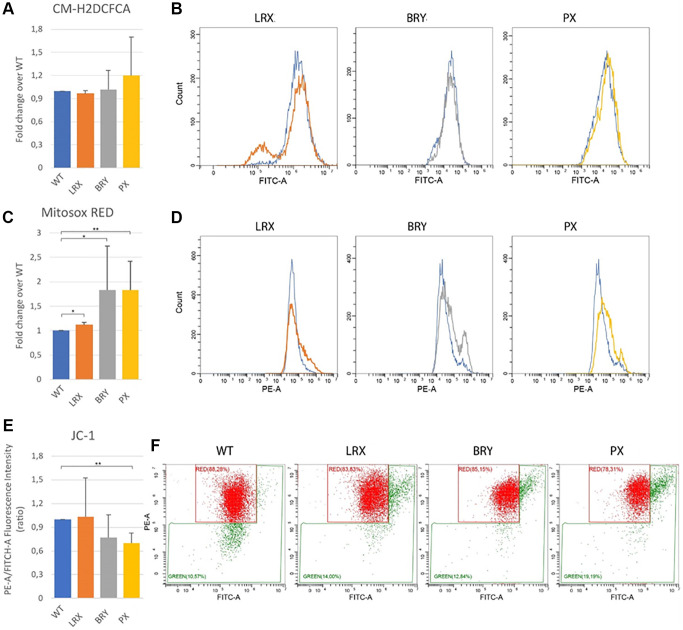
**Mitochondrial dysfunction.** Flow cytometry quantification of total reactive oxygen species using CM-H2DCFDA in MSC WT and MDPL ones. (**A**) Histogram representing the relative fluorescence intensity (FITCH-A), data are shown as mean ± SD of *N* = 3 biological replicates. Adjusted *p*-value was calculated using Student’s *t*-test. (**B**) Representative histogram plots in log scale (WT blue). Flow cytometry quantification of mitochondrial superoxide using MitoSOX Red in MSC WT and MDPL ones. (**C**) Histogram representing the relative fluorescence intensity (PE-A), data are shown as mean ± SD of *N* = 3 biological replicates. Adjusted *p*-value was calculated using Student’s *t*-test. (**D**) Representative histogram plots in log scale. (**E**) Histogram representing the quantification of red/green fluorescence intensity ratio, data are shown as mean ± SD of *N* = 3 biological replicates. Adjusted *p*-value was calculated using Student’s *t*-test. (**F**) Representative dot plots in log scale of JC1 stained MSC WT and MDPL cells. The WT data are derived from the mean of the values obtained from the two healthy control samples; ^*^p < 0.1, ^**^p < 0.01.

Many evidences link telomere shortening to mitochondrial dysfunction [21]. Impaired mitochondria generate excessive reactive oxygen species (ROS), leading to telomere damage. This is why, lastly, we assessed the total ROS production, using chloromethyl derivate of 2′,7′-dichlorodihydrofluorescein diacetate (CM-H2DCFDA) staining and evidencing a trend to increase even though not significant (Figure 8A, 8B). Parallelly the quantification of mitochondrial superoxide, using MitoSOX Red staining, showed a significant increase in all patients, stronger in PX patients (Figure 8C, 8D; ^*^p < 0.1, ^**^p < 0.01). Also, the evaluation of the mitochondrial membrane potential, using JC-1 staining, confirmed the MitoSOX results. The mitochondrial membranes of MDPL cell lines were depolarized, when compared to the mitochondrial membranes of WT cell lines, particularly in PX, where the decrease of the intensity ratio is statistically significant (Figure 8E, 8F; ^**^p < 0.01).

## DISCUSSION

MDPL (OMIM #615381) is a rare (prevalence <1/1,000,000; <30 patients described worldwide) lipodystrophy syndrome caused by dysfunction occurring in a catalytic subunit of DNA polymerase delta, involved in DNA repair and in maintaining genome stability [3]. Patients are clinically characterized by premature ageing together with lipodystrophy, insulin resistance and related metabolic alterations, growth impairment, skin abnormalities and mandibular hypoplasia.

With the aim of deepening the pathogenetic mechanism of the disease, we decided to create MDPL hiPSCs, representing a disease model clinically relevant that allows for the development of precision medicine approaches [22, 23]. MDPL hiPS cells have never been described to date.

The first evidence, as a hallmark of genomic instability or aging, was the presence of micronuclei, exclusively in MDPL hiPSCs. Micronuclei are small nucleus-like structures forming within cytoplasm, separately from the main nucleus [24]. Since Pol-delta is a protein essential for regulating genome stability [25–28], we speculated that micronuclei could represent an expression of this instability in such an early phase of development. In fact, MN formation can be encouraged by agents or genetic perturbations able to enhance either chromosome missegregation in mitosis or to induce DNA damage or replication stress prior to mitosis [29, 30].

We have previously described in MDPL fibroblasts several hallmarks of senescence including inheritable nuclear envelope and/or DNA repair defects and presence of micronuclei [20]. Despite it showing that reprogramming can reset the epigenetic aging clock, it has been established that a residual trace of the donor age could remain in hiPSCs as a partial epigenetic memory [31, 32].

Once labeled for Lamin B1, these MNs appear to be the highest percentage negative for this marker, suggesting the ability of hiPSCs to preserve their fate [33–36]. In fact, in literature it is reported that Lamin B1 prevents the elimination of the MNs, which are certainly a feature of instability [33, 37]. Given the negative impact of MN on genomic and chromosomal stability, the removal of MN or MN-bearing cells could be important for maintaining genomic integrity in cell population.

Successively, hiPSCs were differentiated into mesenchymal cells (MSCs) whose morphological description we immediately focused on, evidencing more micronuclei than those in hiPSCs.

The distribution of Lamin B1 in MSCs is heterogeneous among MDPL, but still in significant proportions. MDPL MSCs showed an increasing trend in respect to MDPL hiPSCs, as if going ahead with differentiation of the cells exacerbates the senescent phenotype. It has to be noted that WT MSCs present a residual quantity of MNs (1.6%), as already described in other cell lines [37, 38], and most of them are Lamin B1 positive, but no senescence signs were evident in these cells.

MSCs typically exhibit a fibroblast-like morphology, characterized by a spindle-shape with a flattened appearance and prominent nuclei. However, MDPL MSCs morphology also reveals a higher prevalence of enlarged nucleus and a spread-out shape, commonly found in senescent cells.

Mesenchymal stem cells (MSCs) are multipotent stromal cells commonly derived from connective tissue. MSCs are present in nearly all tissues, and they can differentiate into osteoblasts, adipocytes, chondrocytes and many other, holding great potential in the field of stem cell-based therapies [39].

In addition, due to the clinical characteristics of our patients, it is challenging to directly sample mesenchymal stromal cells. Therefore, we obtained mesenchymal lines via hiPSCs. According to several studies, the phenotype and function of MSC-hiPSC derived are quite similar to those of conventional bone marrow or adipose-tissue derived MSCs. MSCs derived from hiPSCs can be expanded efficiently, maintaining allogeneic capabilities while being patient-specific [40–43].

MSCs characterization was performed using FACS analysis, labelling these cells with specific markers such as CD73, CD90 and CD44, fulfilling the criteria of MSC markers defined by the ISCT [16–18]. We observed a reduced differentiation efficiency in MDPL MSCs and most importantly an evident individual variability.

Stem cell exhaustion, defined as the decrease in stem cell number and function, is another hallmark of aging. This phenomenon is observed in multiple progeria-related diseases, including Werner syndrome and Hutchinson-Gilford progeria syndrome (HGPS) [44–47]. DNA damage, one of the most important predisposing factors of the aging process, leads to genomic instability [48] and induces in stem cells cellular senescence and exhaustion.

Evaluating other typical hallmarks of cellular aging, we also observed a slowing down of cell growth, an increase of cell death as evidenced also by β-galactosidase activity assay, and a blockage of proliferation in the G0/G1 phase. Moreover, the rate of telomere shortening was statistically and significantly greater in MDPL cells, suggesting the telomere dysfunction as an emerging key feature in MDPL [10]. Our data suggest an accelerated telomere shortening in MSCs of MDPL patients compared to the control group. The reduced telomere length represents one of the characteristics of MSC senescence and results in other progeroid syndromes [49], highlighting their higher susceptibility to DNA damage accumulation [19, 50].

The main cause of DNA damage and telomere erosion, in addition to replication, is the accumulation of reactive oxygen species (ROS) [21, 51]. ROS production is known to be one of the major contributors to the predisposition of aging-related diseases [52, 53]. Imbalanced ROS production and neutralization accelerates oxidative stress, and vice versa, oxidative stress can also induce ROS generation, [54] thus promoting inflammation, cellular senescence, and DNA damage [51]. In MSCs, oxidative stress has been associated with replicative senescence and the reduction of cell potency [55].

Telomere damage leads to mitochondrial biosynthesis dysfunction [56, 57], that in turn causes telomere loss as well as telomere dysfunction-induced foci (TIFs) [58]. An altered mitochondrial activity and telomere erosion were reported both in MDPL fibroblasts [11] and HGPS cells [59–61], as well as a delay in the differentiation process.

Mitochondrial dysfunction is one of the hallmarks of aging [62] and is described in many age-associated diseases, such as Cockayne syndrome [63], Ataxia Telangiectasia [64], Werner syndrome [65], Parkinson’s disease [66], and Alzheimer’s disease [67].

In conclusion, our results, obtained from three MDPL patients, draw a picture of senescent mesenchymal cells with clear morphological abnormalities. These cells differentiate with lower efficiency, proliferate more slowly and have abnormal mitochondrial activity with increased production of ROS. Furthermore, the telomeres show evident shortening. Being senescent, MDPL MSCs exhibit age-related dysfunction, characterized by limitations in their ability to proliferate, and a decline in their stemness properties in generating or restoring new tissue cells in the body [68]. All the characteristics observed in this study show to be heterogeneous among the three patients, and this could be also attributed to several factors, including a different level of Pol δ protein expression.

All these data show that the pathological phenotype at the cellular level becomes evident at a very early stage of human development.

The next step will be to optimize MSCs differentiation into adipocytes and osteocytes, which represent some of the target cells of the disease. Also, multi-omics and single-cell spatial genomic technologies will be performed to verify any differences in diseased cells compared to control cells, and evaluate the response to clinically usable drugs. Other cellular targets involved, such as cochlear inner hair cells or epithelial cells, could be investigated for a deeper and more complete study.

Establishing a connection between cellular senescence and the onset of diseases could shed light on the relevant disease mechanisms. As such, it is critical to understand the mechanisms behind stem cell dysfunction during aging, which impairs tissue function and regeneration ability, thereby contributing to age-related disorders.

## MATERIALS AND METHODS

### Patients

Three MDPL patients were recruited of different ages: BRY (8 years old), LRX (21 years old), PX (32 years old) together with two controls: WT1 (CX: 15 years old) and WT2 (AX: 30 years old).

The two controls essentially always gave comparable results and were therefore often expressed as an average value. Among MDPL patients, BRY sometimes presents a different cellular phenotype that we attributed to his young age and his clinical phenotype which is less severe than the other two. Briefly, he has a triangular face, thin nose with slightly deviated tip, hypoplasia of the nasal wings, hypoplasia of the malar bones, small protruding and folded auricles, high palate, slight retrognathia, small teeth, poor representation of subcutaneous fat, evidence of venous network, and skin xerosis. Diagnosis was made at 7 years old.

Within the context of an evident variability characteristic of Mendelian pathologies, all patients evidently showed skin scleroderma, telangiectasia and subcutaneous lipoatrophy, as well as reduced limb muscle mass. MRI of the abdomen showed an over representation of mesenteric fat.

MDPL patients are heterozygotes for the deletion c.1812_1814delCTC, p.Ser605del, in the *POLD1* gene. Dermal biopsy was performed, following written informed consent and approval by the institutional review board of the Bioethical Committee of Fondazione PTV, Tor Vergata Hospital, according to the principles expressed in the Declaration of Helsinki (protocol n. 2932/2017) [14].

### Differentiation of human pluripotent stem cells (hiPSCs) into mesenchymal stem cells (MSCs)

To differentiate hiPSCs into MSCs, we modified a previously published protocol of Aghadi and collaborators [16]. hiPSCs were dissociated with collagenase IV/Accutase (Sigma-Aldrich, St. Louis, MO, USA) solution into small clumps and transferred on ultra-low attachment plates to form embryoid bodies (EBs). They were cultured in MSC medium (DMEM Low glucose (Gibco, Waltham, MA, USA)), supplemented by 15% FBS (Gibco), 1% P/S (PAA, The Cell Culture Company, Minneapolis, MN, USA) and 1% Gluta-Max (Gibco). At day 2, the medium was changed with the addition of 10 µM retinoic acid (RA), while at day 4 of differentiation the medium was supplemented with 0.1 uM RA. Subsequently, at day 6, the media were replaced without RA until day 8, when the EBs were plated onto pre-coated Matrigel GFR (Corning) plates. At this stage, the medium was changed daily until the spreading produced by the EBs reached approximately 80% confluence. At this point, the cells can be passaged into T25 flasks and cultured in MSC medium containing 2.5 ng/ml bFGF (PeproTech, Thermo Fisher Scientific, Waltham, MA, USA).

The MSC identity and phenotype of the differentiated cells were checked by flow cytometry analysis of MSC markers (CD44, CD73, CD90). The MSCs were cryopreserved in FBS containing 10% DMSO. All the *in vitro* experiments were performed comparing cells at same cell doubling (from passage 3 to 5).

### Flow cytofluorimetry

MSCs were detached from culture plates using trypsin-EDTA solution and washed with phosphate-buffered saline (PBS). Cells were then resuspended at a concentration of 1 × 10^6^ cells/ml in staining buffer (PBS with 2% FBS). Cells were then incubated with anti-CD44-FITC, anti-CD90-PE, and anti-CD73-APC (REAfinity^™^, Miltenyi Biotec, Germany) for 30 minutes at 4°C, protected from light. Isotype controls were included for each antibody. After staining, cells were washed twice with staining buffer to remove unbound antibodies. Cells were then resuspended in staining buffer and acquired with a FacsCanto II flow cytometer. A minimum of 10,000 events per sample were acquired. Flow cytometry data were then analyzed using the software FlowJo version 9.3.2 (BD Life Sciences, Franklin Lakes, NJ, USA). Cells were gated on forward and side scatter characteristics to exclude debris and aggregates. The percentage of cells positive for CD44, CD90, and CD73 was determined relative to the appropriate isotype controls. To ensure the absence of hematopoietic cell contamination, cells were also stained with antibodies targeting hematopoietic markers such as CD34, CD45, CD14 and CD19.

### Western blot analysis

MSCs, grown in T25 flasks, were collected with 0.05% trypsin-EDTA (0.05% Trypsin, 0.53 mM EDTA, 1X; Corning Inc., Corning, NY, USA) when they reached 75% confluence and rinsed with ice-cold DPBS (Dulbecco’s Phospate-Buffered Saline without calcium and magnesium, Corning, REF 21-031-CV) twice. After centrifugation, pellets were lysed in RIPA-Buffer (Pierce™ RIPA Buffer, REF 89901) plus a cocktail of protease inhibitors (Halt™ Protease Inhibitor Cocktail 100X, Prod. #1862209, Thermo Fisher Scientific). Proteins were separated by 4–12% SDS-PAGE gel (NW04125BOX, Thermo Fisher Scientific), then nitrocellulose membranes (88018, Thermo Fisher Scientific) were probed with primary antibodies. The primary antibodies used are: anti-POLD1 antibody (diluted 1:500, ab225907, Abcam, UK), and GAPDH (diluted 1:3000, REF MA5-15738, Thermo Fisher Scientific), used as loading control. Horseradish peroxidase-conjugated secondary antibodies were used: Goat anti-rabbit (diluted 1:1000, REF 32460, Thermo Fisher Scientific) and goat anti-mouse (diluted 1:10 000, REF 31430, Thermo Fisher Scientific). Proteins were visualized with ECL (RPN2235, Cytiva, USA) and chemiluminescence signals were detected using the ChemiDocTM Imaging System (Bio-Rad, Hercules, CA, USA). Densitometric analyses were performed using Image Lab software. The differences between groups were tested by one-way ANOVA test. Values provided in the figures are means of two independent experiments ± standard deviation (SD). The level of significance was established at ^*^p < 0.05, ^**^p < 0.01, ^***^p < 0.001.

### Cell count

MSCs-MDPL and MSCs-WT were seeded at a density of 50,000 cells per well (*n* = 4 wells), in complete growth medium and incubated at 37°C, 5% CO_2_ for 96 h. After 24, 48, 72 and 96 h, cells were tripsinized and counted with Trypan Blue (Gibco) using a Burker chamber. The differences between groups were tested by one-way ANOVA test. Values provided in the figures are means of three independent experiments ± standard deviation (SD). The level of significance was established at ^*^p < 0.1, ^**^p < 0.01.

### MTS assay

MSCs-MDPL and MSCs-WT were seeded at two different densities, 5,000 and 10,000 cells per well, in a 96-well plate. After 48 hours of incubation (37°C, 5% CO_2_), 20 µl of CellTiter 96^®^ AQueous One Solution Reagent (Promega, Madison, WI, USA), containing a novel tetrazolium compound (3-(4,5-dimethylthiazol-2-yl)-5-(3-carboxymethoxyphenyl)-2-(4-sulfophenyl)-2H-tetrazolium, inner salt; MTS) and an electron coupling reagent (phenazine ethosulfate; PES), was added to each well, and the plate was incubated again. After 4 hours, the absorbance of the formazan solution was measured at 490 nm using a spectrophotometer. The differences between groups were tested by one-way ANOVA test. Values provided in the figures are means of three independent experiments ± standard deviation (SD). The level of significance was established at ^*^p < 0.1, ^**^p < 0.01.

### Immunofluorescence staining

Cells grown on coverslips were fixed in 100% methanol at −20°C for 7 min and incubated with appropriate primary antibodies against anti-Lamin A/C (N18; 1:100, Santa Cruz Biotechnology, Inc., USA), anti-Lamin B1 (C-20; 1:100, Santa Cruz Biotechnology, Inc.) and specific Alexa Fluor 568 and 488-labeled secondary antibodies (Invitrogen, Carlsbad, CA, USA) in the presence of Hoechst 33342 (Sigma-Aldrich). Slices were analyzed under a fluorescence microscopy and images are acquired using a Zeiss (Zeiss, Thornwood, NY, USA) Axioplan 2 microscope and Leica TCS SP5 confocal microscope (Leica, Wetzlar, Germany). The differences between groups were tested by one-way ANOVA test. Values provided in the figures are means of three independent experiments ± standard deviation (SD). The level of significance was established ^*^p < 0.1, ^**^p < 0.01.

### Cell cycle analysis by flow cytometry

Cells were harvested during the logarithmic growth phase and fixed in ice-cold 70% ethanol while gently vortexing. Fixed cells were stored at −20°C for at least 1 hour or overnight. Fixed cells were washed with phosphate-buffered saline (PBS) to remove residual ethanol and then resuspended in staining buffer containing propidium iodide (PI: 50 µg/ml) solution supplemented with RNase. Cells were stained for 30 minutes to 1 hour at room temperature in the dark. Cells were then acquired with a FacsCanto II flow cytometer and a minimum of 10,000 events per sample were acquired. Flow cytometry data were then analyzed using the software FlowJo version 9.3.2 (BD Life Sciences). Differences in fluorescence intensity were used to determine the percentage of cells in each phase of the cell cycle (G0/G1, S, and G2/M) after gating on single cells.

### Senescence-associated β–galactosidase staining

SA-β-gal activity assay was performed according to the manufacturer’s protocol (BioVision Senescence detection kit, K320-250). Briefly, MSCs were seeded on glass coverslips, fixed with fixing solution for 15 minutes at room temperature and stained overnight at 37°C with the staining solution (X gal). Images were acquired by Zeiss microscope. The differences between groups were tested by one-way ANOVA test. Values provided in the figures are means of three independent experiments ± standard deviation (SD). The level of significance was established ^*^p < 0.1, ^**^p < 0.01.

### Telomere length analysis

Genomic DNA was extracted from MSCs. Telomere length was measured using the protocol described by Cawthon [69]. The Ct values were determined in each sample during the qPCR run. Relative telomere length was estimated by comparing the amount of telomere repeat amplification product (T, TEL) to a single copy gene (S, β-globin) product. The T/S ratio provided a relative measure of telomere length relative to the reference gene, facilitating comparisons between samples and experimental conditions. The differences between groups were tested by one-way ANOVA test. Values provided in the figures are means of three independent experiments ± standard deviation (SD). The level of significance was established at ^***^p < 0.001, ^****^p < 0.0001.

### Total and mitochondrial ROS production

The cells were then stained with DMSO and CM-H2DCFDA (10 µM; Invitrogen), MitoSOX Red (5 µM; Invitrogen) and JC1 (2 µM; Invitrogen). To allow the permeabilization of the probes, the cells were incubated for 15 min at 37°C, then washed with PBS and analyzed by flow cytometry (Beckman Coulter, Cytoflex) acquiring 10000 events per sample. The CM-H2DCFDA fluorescent signal was collected in the FITC channel, MitoSOX Red in the PE-A channel and for JC1, the signal was collected in both FITC and PE-A channel. The differences between groups were tested by student’s *t*-test. Values provided in the figures are means of three independent experiments ± standard deviation (SD). The level of significance was established ^*^p < 0.1, ^**^p < 0.01.

### Quantification and statistical analysis

Cellular, molecular and biochemical analyses were performed in technical duplicates/triplicates and data were analyzed using GraphPad Prism 8 and the SPSS program, version 25 (IBM Corp, Armonk, NY, USA).

## Supplementary Materials

Supplementary Data

Supplementary Figures
