# Supplementary material for: When do the pathological signs become evident? Study of human mesenchymal stem cells in MDPL syndrome
Source: Aging (Albany NY). 2024 Nov 26;16(22):13505–25. doi: 10.18632/aging.206159 (PMC11723661; doi:10.18632/aging.206159)
Supplement: Supplementary Data [file aging-16-206159-s001.pdf]

## SUPPLEMENTARY DATA

## RESULTS

### MDPL human induced pluripotent stem cells (hiPSCs) shows genome instability

hiPSCs were generated from three different patients and two healthy donors (WT) and characterized following standard procedures [1] (data not shown).

Morphological analyses unexpectedly revealed 5.2% of nuclei altered with micronuclei exclusively observed in MDPL hiPSCs (Supplementary Figure 1A). In addition, these cells also displayed micronuclei (MN) in 0.75% of PX, 2.7% of LRX and 3.35% of BRY (Supplementary Figure 1A).

These novel data on pluripotent stem cells prompted us to perform a more in-depth characterization. We first double-labeled MDPL hiPS clones with OCT4 antibody and the nuclear marker Hoechst to confirm the presence of MNs in stem cells (Supplementary Figure 1B). We then performed FIB/SEM ultrastructural analyses revealing the nuclear envelope alterations, including aberrant shape and indentations, while confirming the presence of MNs (Supplementary Figure 1B).

To shed light on the origin and stability of the MNs, we labeled MDPL clones with the Lamin B1 antibody. Immunofluorescence analysis revealed that 15% of MNs were Lamin B1-positive in LRX cells, and 19.3% in BRY patients, while no immunoreactivity was found in PX MN (Supplementary Figure 1A, 1B). Our data suggest that the content of Lamin B-negative micronuclei is not replicated during the entire cell cycle, indicating a progressive dispersion and removal of micronuclear content during cell proliferation in our cells to preserve genomic stability [2, 3].

## MATERIALS AND METHODS

### Immunofluorescence staining for nuclear stemness marker OCT4 and anti-Lamin B1 antibodies

Cells grown on coverslips were fixed in 100% methanol at −20°C for 7 min or 4% paraformaldehyde for 20 min at RT and incubated with primary antibodies OCT4 (1:25 Novus Biological, Centennial, CO, USA) and Lamin B1 (C-20; 1:100, Santa Cruz Biotechnology, Inc.). Successively incubated with specific Alexa Fluor 568 and 488-labeled secondary antibodies (Invitrogen, Carlsbad, CA, USA) in the presence of Hoechst 33342 (Sigma-Aldrich). Slices were analyzed under fluorescence microscopy and images were acquired using a Zeiss (Zeiss, Thornwood, NY, USA) Axioplan 2 microscope and Leica TCS SP5 confocal microscope (Leica, Wetzlar, Germany). The differences between groups were tested by one-way ANOVA test. Values provided in the figures are means of three independent experiments ± standard deviation (SD). The level of significance was established $^{*}p < 0.1$.

### Ultrastructural analysis by focused ion beam/scanning electron microscopy (FIB/SEM)

Cells plated in appropriate chamber slides were processed for ultrastructural analyses, using Dualbeam FIB/SEM (Helios Nanolab, FEI, Hillsboro, OR, USA). Upon fixation in 0.5% glutaraldehyde and 2% paraformaldehyde, cells were washed in 0.1 M cacodylate buffer, pH 7.4 at 4°C. Osmium tetroxide (1% in cacodylate buffer) was used to postfix samples for 45 min in the dark. After washes, cells were stained with uranyl acetate at 4°C, in the dark, then gradually dehydrated in a graded ethanol series (from 70% to 100%). Samples were then infiltrated with a 1:1 mixture of ethanol and Epoxy Embedding Medium (Sigma-Aldrich™, Cat# 45359-1EA-F), and then embedded in the same resin. Once polymerized at 60°C for 3 days, embedded cells were mounted on stubs and gold coated in an Emitech K550. Cross-sections from regions of interest were analyzed by Dualbeam FIB/SEM (Helios Nanolab, FEI, Hillsboro, OR, USA). Images were acquired using a through-the-lens detector.

### Chromosomal preparations and karyotypic analyses

When mesenchymal stem cells (MSCs) cultured in 75 $cm^2$ culture flasks (Corning, USA) reached 70-80% confluence, they were treated with 10 μg/ml colcemid (BioWest USA, Inc., Bradenton, FL, USA) for 3 h at 37°C before being harvested using trypsin-EDTA 1X solution (Euroclone S.p.A., Italy). The MSCs were resuspended in a hypotonic solution 0.075 M KCl (Sigma-Aldrich™, USA) and incubated at 37°C for 10 min, then fixed in 3:1 methanol: glacial acetic acid (both from Sigma Aldrich™, USA) at room temperature for 10 min. MSCs were then centrifuged and fixed three times (10 min at room temperature, 10 min at room temperature and overnight at 4°C). Slides were allowed to age for 24 hours (60°C) before the staining with Giemsa (Sigma Aldrich™, USA) and G banding, according to the protocol by Seabright [4] with modifications.

Chromosome analysis was carried out on 20 metaphases. The karyotype was expressed following the guidelines of the International System for Human Cytogenomic Nomenclature (2020) (ISCN2020) [5] using software

dedicated to karyotyping: Ikaros Karyotyping Software, version 6.3.9 (MetaSystems, Germany).

## REFERENCES


1. Spitalieri P, Talarico RV, Murdocca M, Fontana L, Marcaurelio M, Campione E, Massa R, Meola G, Serafino A, Novelli G, Sangiuolo F, Botta A. Generation and Neuronal Differentiation of hiPSCs From Patients With Myotonic Dystrophy Type 2. Front Physiol. 2018; 9:967. https://doi.org/10.3389/fphys.2018.00967 PMID:30100878

2. Okamoto A, Utani K, Shimizu N. DNA replication occurs in all lamina positive micronuclei, but never in lamina negative micronuclei. Mutagenesis. 2012; 27:323–7. https://doi.org/10.1093/mutage/ger082 PMID:22086909

3. Utani K, Kawamoto JK, Shimizu N. Micronuclei bearing acentric extrachromosomal chromatin are transcriptionally competent and may perturb the cancer cell phenotype. Mol Cancer Res. 2007; 5:695–704. https://doi.org/10.1158/1541-7786.MCR-07-0031 PMID:17606478

4. Seabright M. The use of proteolytic enzymes for the mapping of structural rearrangements in the chromosomes of man. Chromosoma. 1972; 36:204–10. https://doi.org/10.1007/BF00285214 PMID:5016007

5. International Standing Committee on Human Cytogenomic Nomenclature. ISCN: An International System for Human Cytogenomic Nomenclature; Karger: Basel, Switzerland. 2020.
