# Supplementary material for: When do the pathological signs become evident? Study of human mesenchymal stem cells in MDPL syndrome
Source: Aging (Albany NY). 2024 Nov 26;16(22):13505–25. doi: 10.18632/aging.206159 (PMC11723661; doi:10.18632/aging.206159)
Supplement: Supplementary Figures [file aging-16-206159-s002.pdf]

SUPPLEMENTARY FIGURES

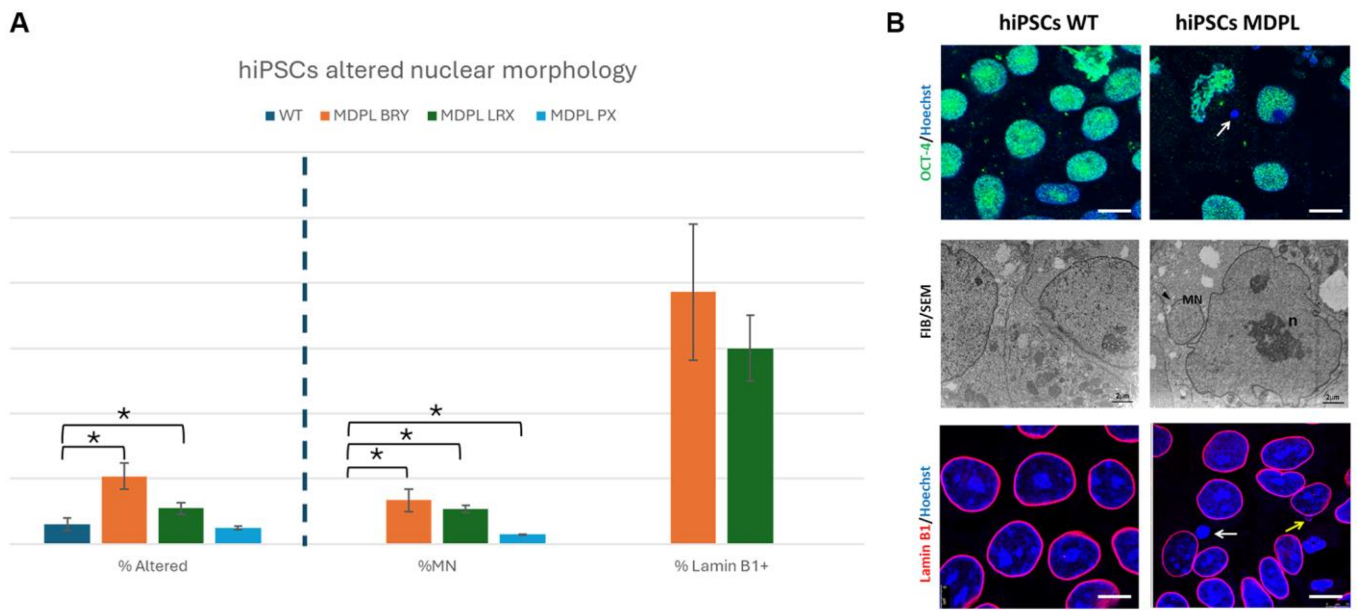

Supplementary Figure 1. hiPSCs altered nuclear morphology analysis. (**A**) Histogram representing the percentage of aberrant nuclear conformations (% altered), percentage of micronuclei (%MN) and percentage of anti-Lamin B1 positive nuclei (%Lamin B1+) in hiPSCs WT and MDPL. (**B**) A representative image of hiPSCs WT and MDPL obtained by immunofluorescence for nuclear stemness marker anti-OCT-4 (green) and anti-Lamin B1 (red) antibodies. Arrows indicate micronuclei staining. Hoechst nuclear staining in blue. FIB/SEM micrographs show abnormal nuclear ultrastructure (n), in MDPL hiPSCs, consisting of irregularly shaped and indented nuclear envelope. Moreover, micronuclei are detected only in MDPL cells. The WT data are derived from the mean of the values obtained from the two healthy control samples. Scale bars = 2 µm and 100 µm. Experiments were carried in triplicates; 100 nuclei were counted per individual. Error bars indicate the standard deviation ± standard error of the mean (SEM). $^{*}p < 0.1$ by one-way ANOVA test.

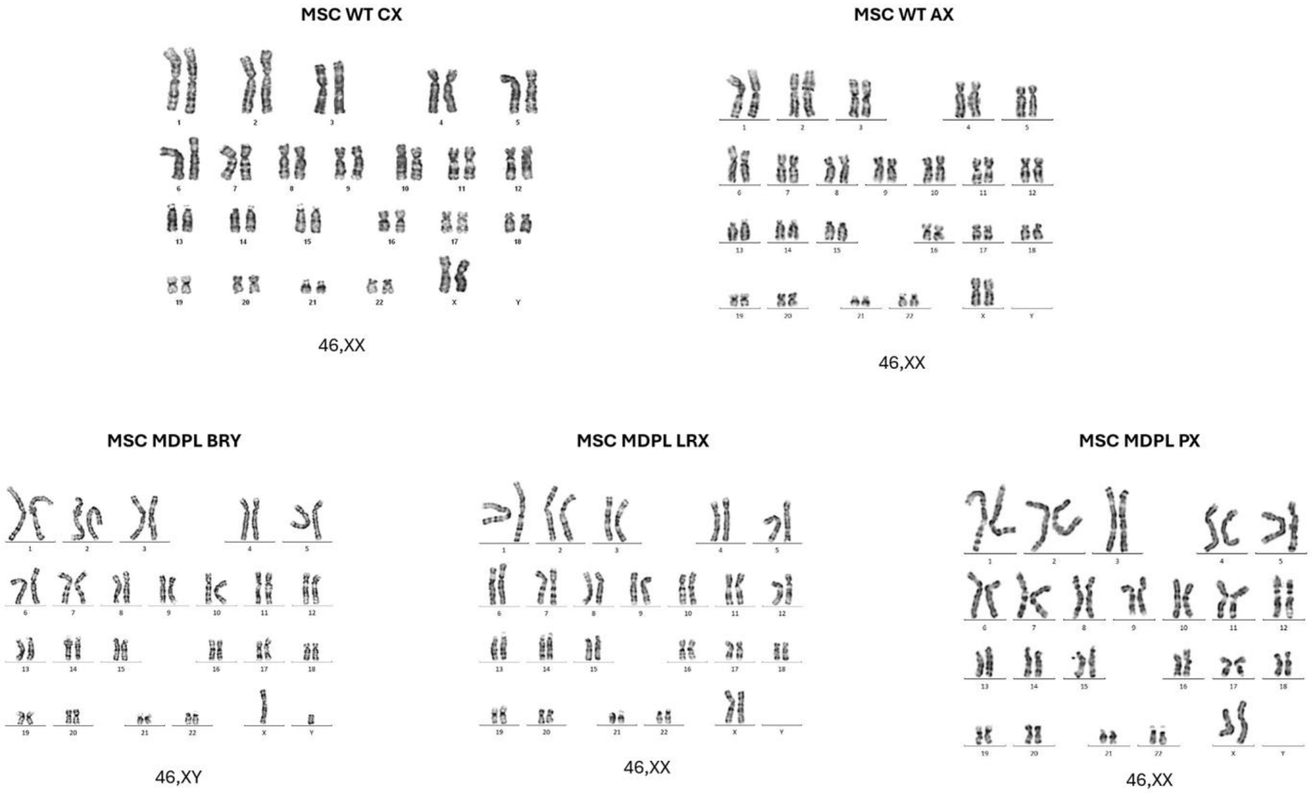

Supplementary Figure 2. Karyotype analysis. G banded analysis of MDPL and WT MSCs metaphase chromosomes revealed a normal karyotype of 46 chromosomes.
